# Isolated giant aortic arch aneurysm repair in a 13-year-old girl: a case report

**DOI:** 10.1186/s13019-023-02311-4

**Published:** 2023-07-01

**Authors:** Volodymyr Vashkeba, Vasyl Karpenko, Oleh Zelenchuk, Sudakevych Serhii, Stepan Maruniak, Justyna Swol, Borys Todurov, Vitaly Demyanchuk

**Affiliations:** 1 Pediatric Department, Heart Institute Ministry of Health of Ukraine, Kyiv, Ukraine; 2 Department of Acquired Heart Valvular Disease, Heart Institute Ministry of Health of Ukraine, Kyiv, Ukraine; 3 Department of Extracorporeal Methods of Treatment, Heart Institute Ministry of Health of Ukraine, Kyiv, Ukraine; 4 Department of Cardiac Surgery, Endovascular and Extracorporeal Technologies, Shupyk National Healthcare University of Ukraine, Kyiv, Ukraine; 5 Department of Respiratory Medicine, Paracelsus Medical University, Nuremberg, Germany

**Keywords:** Congenital heart disease, Aortic arch aneurysm, Aortic arch aneurysm repair, Case report

## Abstract

**Background:**

Aortic arch aneurysm is a very rare condition in children. Surgery is the life saving procedure but it performing might be challenging due to the complex anatomy.

**Case presentation:**

We describe a 13-year-old girl who was diagnosed to have an isolated giant aortic arch aneurysm. This girl was referred to our institution with persistent cough as a leading symptom, which started two months ago. Surgery was performed as combined approach: left-sided thoracotomy and midline sternotomy. The left subclavian artery was re-implanted via supraclavicular approach to the left common carotid artery end-to-side anastomosis. Aneurysm was excised after midline sternotomy and initiation of cardiopulmonary bypass under mild hypothermia. Histological evaluation of the wall of the aneurysm revealed no evidence of any specific changes.

**Conclusions:**

The using of the combined method was characterised by a good postoperative surgical results. Pediatricians should be aware of persistent cough in children as a symptom of mediastinal mass of different origin and identity.

## Background

Aortic aneurysm is a very rare condition in childhood. The pathology may lead to severe complications such as rupture, dissection, or valvular insufficiency [1]. Follow up after aneurysm surgery in the childhood remains difficult, and the optimal time of surgical intervention is not proven.

## Case presentation

A 13-year-old girl (body mass – 45 kg, heigh – 152 cm, body surface area – 1,38 m^2^) was referred to our institution with persistent cough as a leading symptom, which started two months ago. No trauma history, no infection, no genetic diseases (Marfan syndrome, Ehlers-Danlos syndrome, Loeys-Dietz syndrome, familial thoracic aortic aneurysms and dissections, autosomal dominant polycystic kidney disease) or previous surgical interventions were present. Her physical examination was found to be normal. The blood pressure was measured on the right arm 107/60 mm Hg and on the left arm 105/58 mm Hg. Heart rate was 80 beats per minute and peripheral oxygen saturation at 98%.

**Fig. 1:**
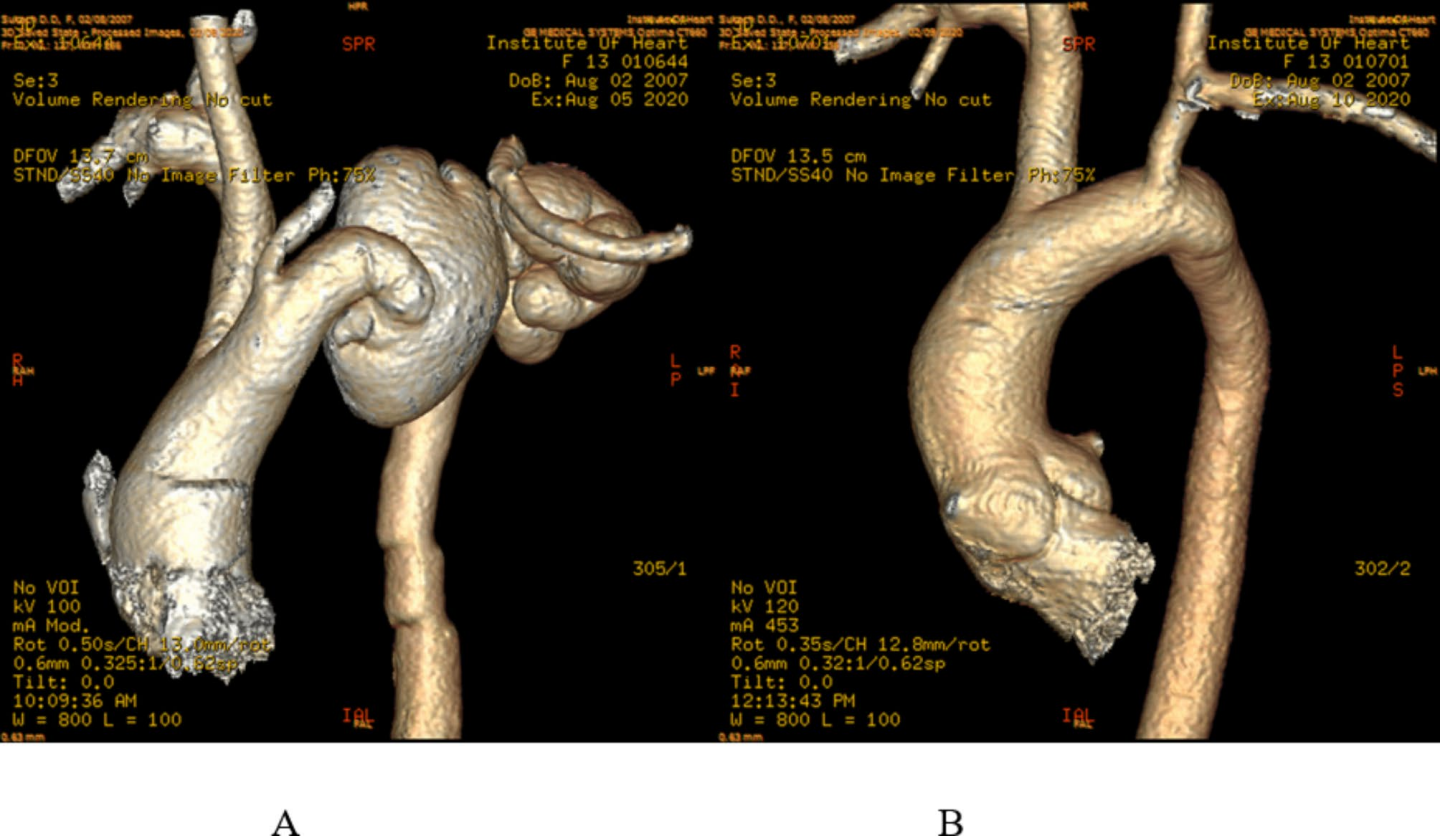
**A**) 3D reconstructed pre-operative CT angiogram; **B**) 3D reconstructed post-operative CT angiogram

Chest x-ray showed asymmetric upper mediastinal mass with a clear outer contour. Echocardiography showed echofree formation with a clear outer contour of 5.0*4.0 cm at the level of the aortic arch, tricuspidal aortic valve. Computed tomography (CT) of aorta visualized distal arch aneurysms up to 5.18*3.63*3.47 cm and up to 4.45*2.20*1.67 cm behind the ostium of the left subclavian artery, ascending aorta – 1,96 cm, aortic root – 2,78 cm, sinotubular junction – 2,37 cm (Fig. 1A).

**Fig. 2:**
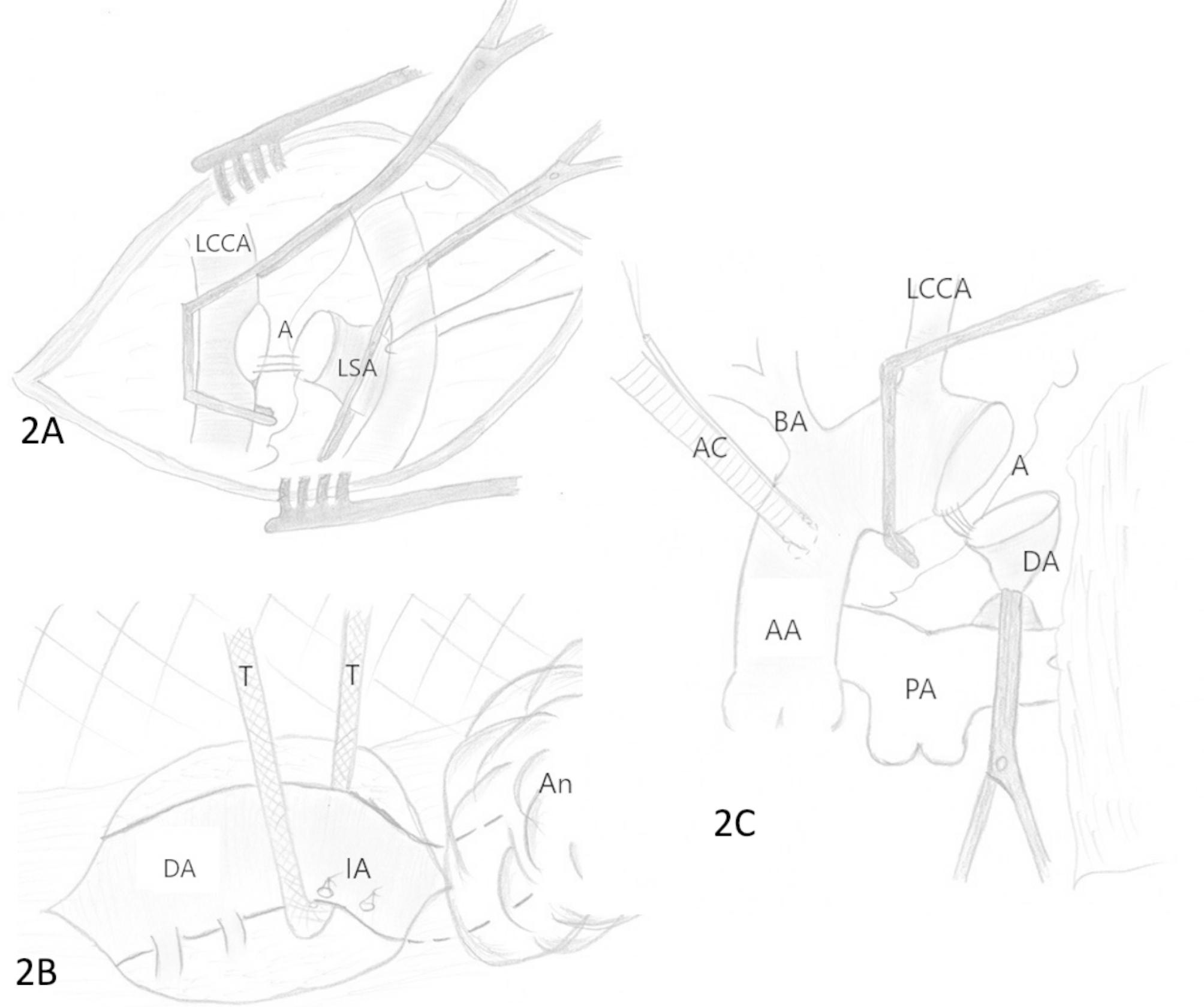
Schematic illustration of intraoperative maneuvers. **A**) Supraclavicular approach and LSA to LCCA end-to-side anastomosis (LCCA = left common carotid artery; LSA = left subclavian artery; A = anastomosis); **B**) Left sided thoracotomy, ligation and transection of intercostal arteries and mobilization of descending aorta (DA = descending aorta; IA = remnants of intercostal arteries; An = aneurysm; T = tape) **C**)Medline sternotomy and aortic end-to-end repair (AA = ascending aorta; AC = aortic cannula; BA = brachiocephalic artery; LCCA = left common carotid artery; DA = descending aorta; PA = pulmonary artery; A = anastomosis)

The patient was taken to the operating room where a combined surgical approach was utilized. Surgery was performed during a single operative session. Firstly, the left supraclavicular approach was performed. The left subclavian artery was dissected, doubly ligated, and divided. It was then connected to the left common carotid artery by means of a native end-to-side anastomosis using a continuous prolene 6/0 stitch (Fig. 2a). The skin in this area was closed. After that, the patient underwent a left-sided thoracotomy through the 4th intercostal space where the descending thoracic aorta was carefully and extensively mobilized within the 3rd and 4th pairs of intercostal arteries with no touch of the aneurysm wall (Fig. 2b). For this reason, 2 pairs of intercostal arteries were transected. A chest drainage tube was inserted in the left hemithorax, and the thoracotomy incision was stitched as well. Then the patient was returned to the supine position on the operative table, and midline sternotomy was performed. After initiation of cardiopulmonary bypass with ascending aorta and right atrium cannulation, the patient was cooled to + 28°C. The aorta was cross-clamped in two separate locations: the first clamp was placed right distally from the brachiocephalic artery, and the second one on the previously prepared segment of the descending thoracic aorta. The pump speed was reduced to 15% according to initial blodflow during CPB, and antegrade cerebral perfusion were started and lasted over 12 min. The aneurysmal sac was punctured, and there was no sign of retrograde blood flow within it. The aneurysm was successfully resected, and the aorta was reconstructed in an end-to-end anastomosis fashion using continuous prolene 5/0 (Fig. 2c).The transected intercostal arteries were pulled toward health part of the aortic arch during the final stage of surgery. The patient was weaned from cardiopulmonary bypass and transferred to the pediatric intensive care unit.“

Histological evaluation of the wall of the aneurysm in our patient revealed no evidence of any specific changes. On CT of aorta on fourth day after the surgery the brachiocephalic trunk and the left common carotid artery (the left subclavian artery is sutured to the left common carotid artery) were identified (Fig. 1B). A small hematoma was persisting in the anterior mediastinum on the left.

The postoperative course was uneventful, and the patient was discharged home on the eleventh post-operative day. No complain was recorded at a routine examination one year after the surgery.

## Discussion and conclusions

Aortic aneurysm repair in children is a challenge due to the low level of evidence of this pathology. In our case report the using of the combined method was characterised by a good postoperative surgical results.

A few articles related to the aortic aneurysms repair in children are existing in the literature (Table 1). Hetzer et al. described a successful surgical treatment of an isolated giant aneurysm of the ascending aorta and its arch in an 8-month-old infant with deep circulatory arrest [2]. The evidence shows, that infant patients are not affected in the same way in deep hypothermic circulatory arrest as teenagers are. Further, aortic aneurysm repair in adolescent may not require replacement of aortic composite grafts, unlike infants who will grow. Another case reported by Caputo et al. was a successful repair of large aortic arch aneurysms in a 15-year-old girl using deep circulatory arrest [3]. Similar to our patient, this girl had no congenital or genetic disease that could have caused the aneurysm, but most likely aortic aneurysm was associated with aortic coarctation.

**Table 1:** Review of literatures

Authors	Publ. year	Age / gender	Procedure	Outcomes
Cattaneo SM, et al. [4]	2004	fifty children (age 1.5 to 18.9 years old)	Surgery for aortic root aneurysm	87% survival at 15 years
Hetzer R, et al. [2]	2008	8-month-old infant	Replacement of an isolated giant aneurysm of the ascending aorta with an adult-sized synthetic graft.	successfully treated
Caputo M, et al. [3]	2015	15-year-old girl	Aortic arch was reconstructed with an interposition graft and re-implantation of the left subclavian artery	successfully treated
Takeshita M., et al. [5]	2019	10-year-old girl	The ascending aorta, aortic arch, and descending thoracic aorta were replaced via median sternotomy and left thoracotomy	successfully treated

A low surgical risk of aortic aneurysm repair in children also was shown by Cattaneo et al., who review their 21-year experience in 50 children who have undergone aortic root replacement using three different operative procedures [4]. This study suggests that good long-term surgical results (87% survival at 15 years) may be achieved with current surgical procedures.

In the recent study Takeshita M described a case involving 10-year-old girl who had an arch and descending thoracic aortic aneurysm. As in our case the aortic aneurysm was replaced via median sternotomy and left thoracotomy with safe systemic and brain perfusion to avoid placing the anastomoses within the inflammatory lesion.

Surgery for aortic aneurysm repair is feasible in adolescent patients. Surgical replacement of aorta was safely performed with the help of preoperative CT findings. Pediatricians should be aware of persistent cough in children as a symptom of mediastinal mass of different origin and identity.

## Data Availability

Data sharing is not applicable to this article as no datasets were generated or analysed during the current study.

## Abbreviations

CT: Computer tomography
